# Transformer-Based Fire Detection in Videos

**DOI:** 10.3390/s23063035

**Published:** 2023-03-11

**Authors:** Konstantina Mardani, Nicholas Vretos, Petros Daras

**Affiliations:** Information Technologies Institute (ITI), Centre for Research and Technology Hellas (CERTH), 57001 Thessaloniki, Greece

**Keywords:** transformers, fire detection, segmentation, real-time, videos, fire localization, image classification

## Abstract

Fire detection in videos forms a valuable feature in surveillance systems, as its utilization can prevent hazardous situations. The combination of an accurate and fast model is necessary for the effective confrontation of this significant task. In this work, a transformer-based network for the detection of fire in videos is proposed. It is an encoder–decoder architecture that consumes the current frame that is under examination, in order to compute attention scores. These scores denote which parts of the input frame are more relevant for the expected fire detection output. The model is capable of recognizing fire in video frames and specifying its exact location in the image plane in real-time, as can be seen in the experimental results, in the form of segmentation mask. The proposed methodology has been trained and evaluated for two computer vision tasks, the full-frame classification task (fire/no fire in frames) and the fire localization task. In comparison with the state-of-the-art models, the proposed method achieves outstanding results in both tasks, with 97% accuracy, 20.4 fps processing time, 0.02 false positive rate for fire localization, and 97% for f-score and recall metrics in the full-frame classification task.

## 1. Introduction

CCTV (closed-circuit television) control systems are always installed in industrial fields to protect the area from either illegal human activities or physical anomalies that could have catastrophic effects. Given the widespread use of these control systems, a number of automatic detection algorithms have been integrated into them to automatically alert in case of danger, such as fire [1]. Fire detection refers to the ability of control systems to identify and detect fire as quickly as possible. It can provide an early warning of fires, helping to minimize property damage and prevent the loss of life. Additionally, fire detection can be integrated into other smart building systems, such as fire suppression systems, to provide a comprehensive fire safety solution [2].

On the first steps of the research in the field, researchers suggested image processing techniques, using handcrafted features extracted from color, texture, contour, motion information, etc., either alone or in combination with machine learning approaches, by using classifiers such as SVMs, random forests, Bayes [3] for the fire detection task in videos. Recent research has made use of deep learning techniques, as they can automatically extract features and provide better results. Many approaches [4,5,6,7] in the literature for the fire detection in videos task use their own video datasets, which are not published for training and evaluation, or image datasets. Others although they attempt to solve the localization problem in videos, they evaluate their performance on the full-frame classification task (the presence of fire in frame or not), without evaluating their localization performance [8,9,10,11] and others prefer to present their results in fire localization task without comparing them with the results of prior works [7]. All these derive from the fact that there is not a complete fire detection in videos benchmark, annotated with segmentation masks in all frames, that contains an adequate amount of videos.

This paper introduces transformers for the fire detection task in videos. A transformer is a model that applies the self-attention mechanism, which measures the significance of each part of the input features (visual features, word embeddings, etc.), according to the problem one attempts to solve, in the form of weighting scores. Doing so, it utilizes the most relevant parts from the input [12]. It was initially applied to natural language processing (NLP) [13], but due to its success in that field, its use extended to computer vision tasks, such as image classification, object detection, and action recognition [12]. It is an encoder–decoder architecture, where the encoder consists of layers generating attention scores that indicate which parts of the input are relevant to each other. The decoder consists of layers that project the features in different and more suitable forms [12]. In NLP, the translation from one language to another is such an example.

The proposed approach is following the transformer from [14], but in a simplified form. This method removes the bipartite matching, class head, and bounding box head because they are unnecessary for the task and replaces the object queries, which are learnable embeddings, with the feature vector of the frame that is under process, in order to reduce model parameters, as this does not reduce the quality of the output. More specifically, both the encoder and the decoder consume the features from the current frame that is under process, in order to generate attention scores. For the fire localization task, these scores pass through a head, called mask head, that produces segmentation masks in the same way with [14]. Following [14], the mask head consists of a multi-head attention layer, in order to seek visual correlations between the features of the encoder–decoder outputs and a FPN architecture module for increasing the resolution of the predicted segmentation mask. For the full-frame classification task, the decoder attention scores pass through a linear layer, in order to predict the frame class (fire/no fire).

For the needs of the proposed method for the fire localization task, a segmentation mask dataset was created, based on the [15] dataset, by applying the SLIC [16] method, which divides the frame by superpixels. The superpixels that are totally enclosed in the ground truth bounding box rectangle were considered fire superpixels, and all the others were considered non-fire. This produces segmentation masks that specify the exact location and shape of the fire in the frames, according to the ground truth bounding boxes.

This study is divided into five sections. Section 1 highlights the need of integrated fire detection systems in surveillance systems, and the importance of accurate and fast models for their reliable performance. Additionally, the methodology is introduced and some prior works are referenced. Section 2 presents two categories of prior works in more detail: methodologies based on handcrafted features and deep learning-based methods. Section 3 describes, in detail, the proposed method, module by module. Section 4 presents the quantitative and qualitative results of the proposed method, in comparison with prior works, the implementation details, and discussion of the results. Finally, in Section 5, the study and its findings are summarized.

## 2. Related Work

As can be seen in the literature, the first efforts for fire detection in videos focused on the extraction of handcrafted features related to color, texture, motion information, etc, and their analysis, which provided remarkable results. However, deep learning-based approaches have gained field, due to their top performance.

### 2.1. Handcraft Features

In 2007, Celik et al. [17] used a generic color model in order to propose a fuzzy logic-based fire detection method. YCbCr color space was chosen for its deterministic characteristics in the Y, Cb, Cr color channels. For example, in a fire pixel, it is more possible that Y(x,y) will be greater than Cb(x,y) because the luminance information, which is related to the intensity of the pixel, is expected to be high. Three years later, Celik et al. proposed a combined color- and motion-based fire detection method, but this time in the CIE L*a*b* color space, in order to extract color related features. They used a threshold-based classifier in order to subtract background from fire regions [18]. The same year, Zhou et al. [19] proposed a contour-based fire detection problem, which consists of three stages: (1) the candidate fire frame selection stage to select the most suspicious frames and remove the others, (2) the fire region selection stage to detect the fire pixels by fire-region selection rules, and (3) the contour-based fire decision, where it performs four operations (dilation, erosion, mini region erasing, and Canny edge detection) on all fire regions, in order to detect the exact fire contours, and finally, fire decision rules based on three characteristics (i.e., area, perimeter, and roundness of the flame contours) to determine whether a fire occurs in the video or not. The next year, Chenebert et al. [20] proposed a non-temporal texture-driven method for fire detection, where they used texture- and colour-based feature descriptors as input to train classifiers, such as neural networks and regression trees. For texture descriptors, they used hue and saturation from the HSV color space. This model provided competitive results, until now. A fire detection method based on shape variation, color, and motion was proposed by Foggia et al. [21], which combined with a multi-expert system and weighted voting, in order to manage the high dimensional feature vectors derived from the color and motion characteristics of fire. Additionally, they proposed a descriptor based on a bag-of-words approach for the motion representation. More recently, Kong et al. [11] proposed the use of logistic regression and temporal smoothing. More concretely, for the determination of a candidate fire region, they took into consideration the color component ratio, and they obtained the motion cue of fire from background subtraction. For size, motion, and color information, they used logistic regression. In order to differentiate fire, fire-like objects, and background, they used distribution and Chroma ratio (Cb/Cr). They concluded that temporal smoothing reduces false alarms. Han [10] proposed the use of motion and color features for their fire detection approach. They used the Gaussian mixture model for background subtraction and a combination of RGB, YUV, and HSI color spaces for the multi-color detection. With the help of these two steps they identify the fire areas in the image plane. Gong et al. [22] also proposed motion and color features for their fire detection method, but they combined a motion detector and a RGB color model to screen the candidate fire pixels. They proposed a fire centroid stabilization method based on spatio-temporal relation, in order to calculate the centroids of fire in all frames and leverage this temporal information for the fire localization task, after passing through a support vector machine.

### 2.2. Deep Learning Approaches

Although many deep learning models have been introduced for the detection and localization of fire regions in videos, there are few that specify this region in the pixel-level classification form, due to the lack of adequate datasets both for training and evaluation. In 2016, Zhang et al. [23] proposed a CNN-based method for forest fire detection by passing through the fire classifier image patches. The approach starts by checking for fire presence in the full image, in order to continue to the next step, which localizes the image patches in the image plane. They also built a fire detection dataset with patch-level annotations. In 2018, Zhao et al. [24] attempted to tackle the difficulties that arise when the frames are obtained from moving UAVs (Unmanned Aerial Vehicles). They proposed a saliency detection method combined with a logistic regression classifier. The saliency detector extracts the region of interest in which the presence of fire is possible, and then two logistic regression classifiers specify if the region of interest forms a fire region or smoke region using color and texture features. Their method prevents feature losses by using fixed image size for training. In 2019, Aslan et al. [5] proposed deep convolutional generative adversarial neural networks (DCGANs) for the fire detection in videos. Their approach demands two-stage training. In the first stage, they train spatio-temporal images (temporal slice images) and noise vectors, and in the second stage, they train the discriminator without the generator. The same year, Yu and Chen [6] proposed a method that consists of three stages: In the first stage, it preprocesses the video by combining motion and color feature detection, in order to extract fire regions. Therefore, the suspected region passes through a spatial convolutional neural network, and the stacked optical flow of the fire region passes through a temporal convolutional neural network. Finally, their results were fused to give the ultimate prediction. Muhammad et al. [4] proposed a computational efficient CNN architecture inspired by the SqueezeNet, in order to be more easily applicable in real-life situations. They used smaller convolutional kernels and no dense or fully connected layers at all. However, they achieved comparable results with other architectures (AlexNet, VGG). In 2018, Dunnings and Breckon [25] experimented with three low-complexity CNN architectures (AlexNet, VGG-16, InceptionV1) providing, as input, superipixels extracted from frames to be classified as fire or non-fire. The InceptionV1 model provided the best results. Next year, Samarth et al. [9] proposed InceptionV3 and InceptionV4, inspired by InceptionV1, ResNet, and EfficientNet, with modifications in kernel sizes and the number of convolution layers. InceptionV4 produced the most accurate results. Another approach based on superpixels was proposed in 2020 by Thomson et al. [8], but this time, they experimented with NasNet-A-Mobile and ShuffleNetV2 architectures, but in a simplified form. ShuffleNetV2 gained performance compared to all other superpixel methods. Kim and Lee [7] introduced faster region-based convolutional neural networks (R-CNNs) for detecting possible fire regions based on their spatial features. The features included within the bounding boxes of sequential frames are aggregated and pass through a long short-term memory (LSTM) for classifying them as fire or not fire in a short-term period. The final decision arises from a majority voting after combining the obtained short-term decisions.

## 3. Materials and Methods

**Figure 1:**
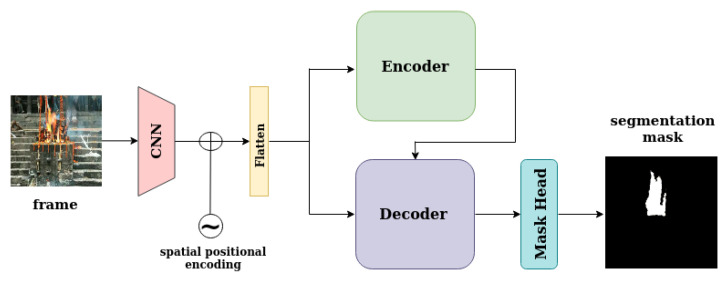
This figure depicts model architecture for the in-frame fire localization task. Both the encoder and the decoder consume the current frame that is under examination and output attention scores. These attention scores pass through the mask head in order to produce segmentation mask that localizes fire in the image plane.

**Figure 2:**
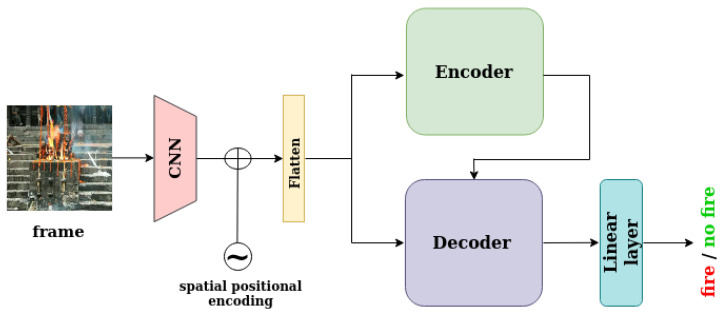
This figure depicts model architecture for the full-frame classification task. Both the encoder and the decoder consume the current frame that is under examination and output attention scores. The output of the decoder pass through a linear layer in order to predict the frame class (fire/no fire).

The proposed method introduces a fire detection method for videos based on transformers. The transformer encoder consumes the features of the frame that are under examination and outputs attention scores that indicate the correlations between the features of the same frame. The transformer decoder consumes features from the current frame, as well as the output of the encoder, in order to compute attention scores between the two representations. For the fire localization task, on top of the decoder, there is a mask head, which predicts a segmentation mask that localizes and describes the fire in the current frame, as can be seen in Figure 1. For the full-frame classification task, on top of the decoder, there is a class head with a linear layer for binary classification (fire/no fire), as can be seen in Figure 2. Therefore, the model consists of three components: a convolutional backbone, a transformer encoder–decoder, and a mask head for predicting segmentation masks or a linear layer for predicting the frame class.

### 3.1. Encoder/Decoder Backbone

**Figure 3:**
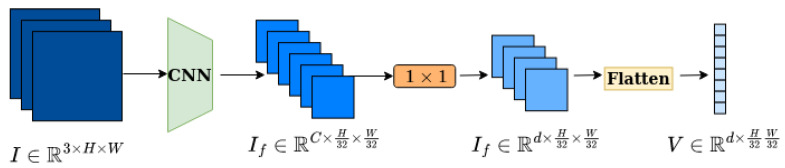
In this figure the encoder/decoder input pipeline is depicted. Both to the encoder and to the decoder, the frame passes through the same transformations until it is fed into the encoder/decoder blocks. Encoder and decoder inputs have the same dimensions.

The model uses ResNet-101 to extract features from frames, in order to pass them through the encoder/decoder. It is fed with a frame $I\in R^{3\times H\times W}$, and it generates a feature map $I_{f}\in R^{C\times \frac{H}{32}\times \frac{W}{32}}$, where *H* and *W* are the height and width of the input frames, respectively. The output of the backbone then passes through an 1×1 convolutional layer to reduce the number of channels from *C* to *d* (C=2048,d=128) for computational efficiency. As both the encoder and the decoder expect sequences for input, the feature map is flattened to a feature vector $V\in R^{d\times \frac{H}{32}\times \frac{W}{32}}$ as can be seen in Figure 3.

### 3.2. Transformer Encoder

**Figure 4:**
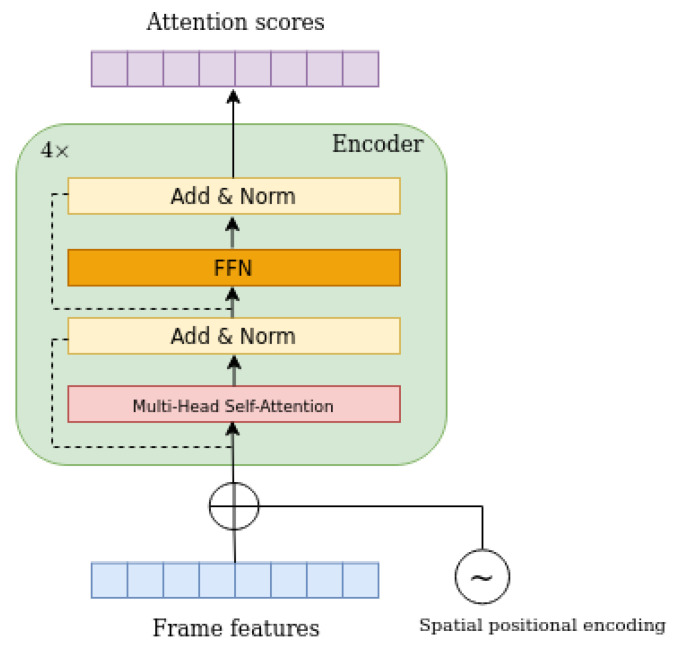
In this figure, the encoder layer architecture is depicted. The encoder of the model consists of four identical, consecutive layers.

Similarly to the architecture of the transformer in [14], each encoder layer consists of a multi-head self-attention module with *M* heads, where M=8, and a feed forward network (FFN). The encoder consists of *N* identical encoder layers, where N=4, as can be seen in Figure 4. Due to the fact that the transformer architecture is permutation-invariant, a fixed positional encoding [26,27] is added both to the feature vector and to the input of each attention layer. The output of the encoder is attention scores with the same shape as its input, but in different representation.

### 3.3. Transformer Decoder

**Figure 5:**
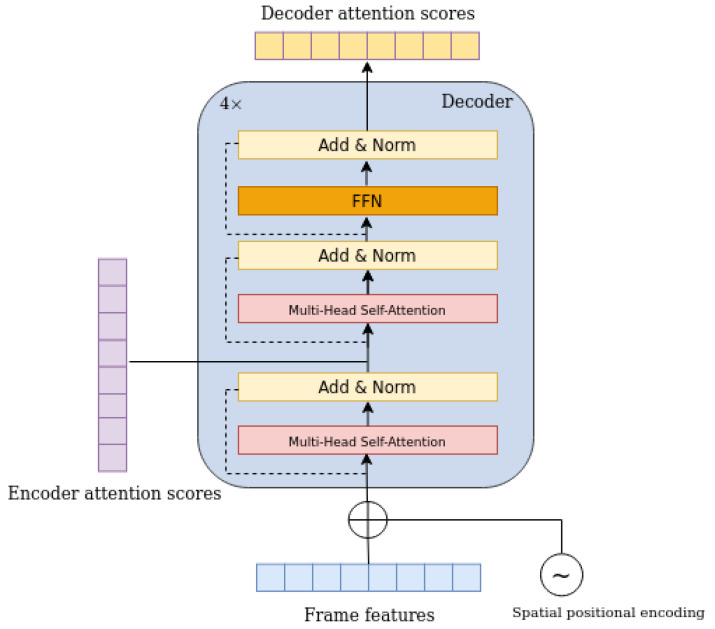
In this figure, the decoder layer architecture is depicted. The decoder of the model consists of four identical, consecutive layers.

The same feature vector with the encoder passes through the decoder. Each decoder layer consists of two multi-head self-attention layers with *M* heads, where M=8, and a feed forward network (FFN). The decoder contains four identical and consecutive decoder layers. Due to the fact that the transformer architecture is permutation-invariant, a fixed positional encoding [26,27] is added both to the feature vector and to the input of each attention layer. Additionally, the decoder consumes the obtained encoder attention scores, as can be seen in Figure 5, which pass through a multi-head self-attention layer fused with the decoder input, in order to obtain visual correlations between the two representations.

### 3.4. Fixed Positional Encodings

Positional encodings in computer vision, are a method used in the transformer architecture to allow the model to understand the relative position of elements in an image. The fixed positional encodings are pre-calculated, numerical values that are added to the input representation of each element in the image. These values encode the relative position of the elements, allowing the model to distinguish between elements that are near or far from each other. The fixed positional encodings are learned during the training process and are used to enhance the representation of the input image, thus improving the accuracy and performance of the model. They are added to the input representation before the model begins processing the data, allowing the model to take into account the relative position of the elements in the image. In the proposed method, the sinusoidal positional encodings are used. This encoding method involves adding a sinusoidal function to the image elements, based on their position in the image [13,28].

### 3.5. Mask Head

**Figure 6:**
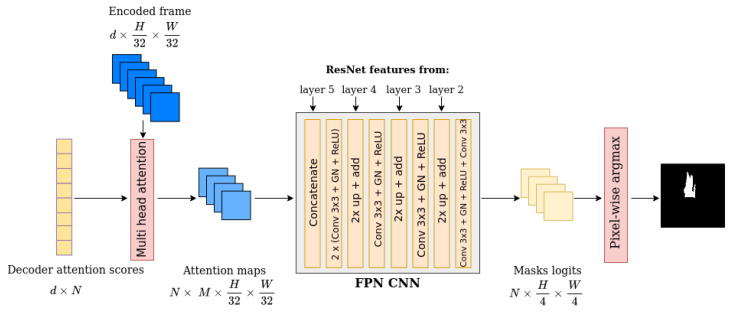
In this figure, the mask head architecture is depicted. All the steps from the decoder attention scores, until the final segmentation mask, are illustrated.

For the fire localization task, on top of the decoder output, the class head and the bounding box head that DETR [14] uses for predicting bounding box coordinates and object classes are removed. These two heads are unnecessary in the proposed method for the localization task, as the model predicts only segmentation masks. For the prediction, the output of the decoder and the output of the encoder pass through a multi-head attention layer with *M* heads, where M=8, to seek correlations between their features. This generates *M* attention heatmaps in low resolution. For the increase of the resolution for the final prediction an FPN (feature pyramid network) is used, as can be seen in Figure 6.

### 3.6. Class Head

For the full-frame classification task, on top of the decoder output, the mask head and the bounding box head that DETR [14] uses for predicting bounding box coordinates and panoptic segmentations are removed. These two heads are unnecessary in the proposed method for the classification task, as the model predicts only the frame class (fire/no fire). For the prediction, the output of the decoder pass through a linear layer, which is a fully connected layer (FCN), where every input neuron is connected to every output neuron. The number of the input neurons is 128, and the number of output neurons is 2, equal to the number of classes (fire/no fire).

### 3.7. Losses

#### 3.7.1. Fire Localization

This proposed method for fire localization is trained using for loss function the combination of *DICE loss* and *focal loss*, similarly to the DETR [14] for panoptic segmentation. The loss function can be written as:

(1) $$L=L_{DICE}+L_{focal}$$

*DICE loss* [29] is a measure of overlap between two sets. In computer vision, it is widely used to measure the similarity between two images. If *p* is the prediction binary mask and *t* is the target mask, $L_{DICE}$ is defined as:

(2) $$L_{DICE}=1-\frac{2t\sigma (p)+1}{\sigma (p)+t+1}$$

where σ is the sigmoid function.

*Focal loss* [30] on the other hand addresses the class imbalance, by downweighting the contribution of the easy examples during training, while focusing the model’s attention on hard examples.

(3) $$L_{focal}=-{\left(1-p_{c}\right)}^{\gamma }log\left(p_{c}\right)$$

where $p_{c}$ is the predicted class probability and γ is the focusing parameter (γ=0.25).

#### 3.7.2. Full-Frame Classification

The proposed method for full-frame classification is trained using for loss function the cross-entropy loss, the most commonly used loss function for categorical models.

(4) $$L_{CE}=-\sum_{c=1}^{n}t_{c}log\left(p_{c}\right)$$

where *n* is the number of classes (n=2), $t_{c}$ is the ground-truth, and $p_{c}$ is the probability of the *c*th class.

## 4. Experiments

In this section, the experimental results are presented. In the first subsection, the datasets and metrics that have been used for training and evaluation are detailed. Subsequently, the implementation details and the qualitative results are portrayed, and finally, the quantitative results are compared with the state-of-the-art methods.

### 4.1. Datasets

For the fire localization task, the model has been trained and evaluated on the furg fire dataset [15], the only video-based fire detection benchmark that consists of fire and non-fire image sequences and bounding box annotations for each frame. This dataset contains 24 videos published on the internet with 28,022 frames, under the Creative Commons 3.0 license. For the training and the evaluation of the proposed method, the dataset is split into training and testing sets, similarly to in [8,9,25]. The used dataset consists of 26,339 full-frame images with 14,266 fire images and 12,073 non-fire images. The training set consists of 18,590 images and the testing set consists of 2211 images.

Additionally, the proposed method has been trained and evaluated on the dataset from work [25] for the full-frame classification task. It is a combination of the [20] dataset, the [25] dataset, and videos from public sources (YouTube).

It contains 26,339 images, with 14,266 images of fire and 12,073 of non-fire images. The training set consists of 23,408 images, and the testing set consists of 2,931 images.

### 4.2. Evaluation Metrics

For evaluation purposes, the proposed method was compared to prior works based on their ability to address two different problems: (a) the full-frame classification problem (if fire exists in the frame or not) and (b) the fire localization within the frame problem (predicting the exact location of fire in the image).

#### 4.2.1. Full-Frame Classification

For the evaluation of the proposed method on the full-frame classification problem, precision (P/PPV), true positive rate or recall (TPR), F1-score (F1), false positive rate (FPR), accuracy (A), and frames per second (fps), according to [15], were used to compare the different methods on two different datasets.

#### 4.2.2. Fire Localization

For the evaluation of the proposed method on the in-frame localization task, precision (P/PPV), true positive rate or recall (TPR), F1-score (F1), similarity (S), and frames per second (fps), according to [15], were used to compare the different methods.

### 4.3. Implementation Details

**Figure 7:**
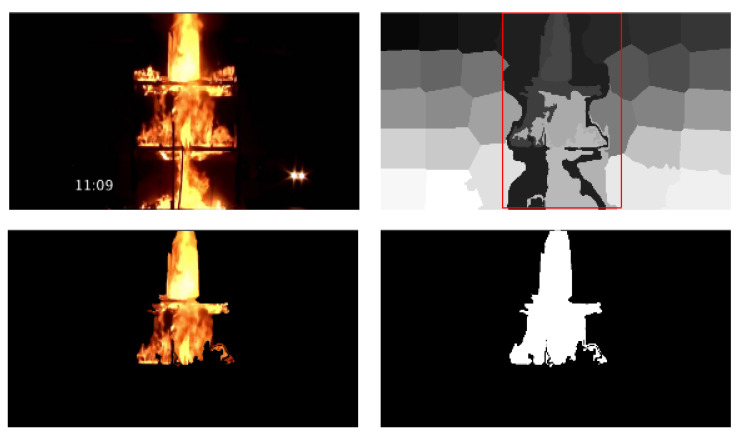
This figure illustrates the processing steps, in order to obtain the segmentation dataset from the bounding box annotations.

For the needs of the proposed method for the fire localization task, a segmentation mask dataset was created, based on [15]. A method called SLIC [16], which generates superpixels by clustering pixels based on their color similarity and their proximity in image plane, was applied at each frame, in order to divide it into superpixels. The superpixels that were entirely included into the ground truth bounding box rectangle (red box in the top right image of Figure 7) were labeled as fire (1), and all the other superpixels were labeled as non-fire (0). This pipeline is depicted in Figure 7. This produces segmentation masks that specify the exact location and shape of the fire in the frames, according to the ground truth bounding boxes, as can be seen in Figure 7.

The proposed method was implemented in Pytorch 1.5.1 version [31] and trained using AdamW optimizer [32], with a weight decay at 0.0001, learning rate at 0.00001, and learning rate for backbone at 0.00001 too. The backbone was an ImageNet-pretrained ResNet-101 model [33] with frozen batchnorm layers. The input size was set to 480×854, and the batch size to 1. For the in-frame fire localization task, the probability threshold for producing the segmentation mask was set to 0.6. This means that, if a pixel had probability higher than 0.6, it was labeled as fire pixel, else as a non-fire pixel. The model was trained on GeForce RTX 2070 and achieved top results on epoch 6 for both tasks.

### 4.4. Evaluation Results and Discussions

#### 4.4.1. Fire Localization Model

**Figure 8:**
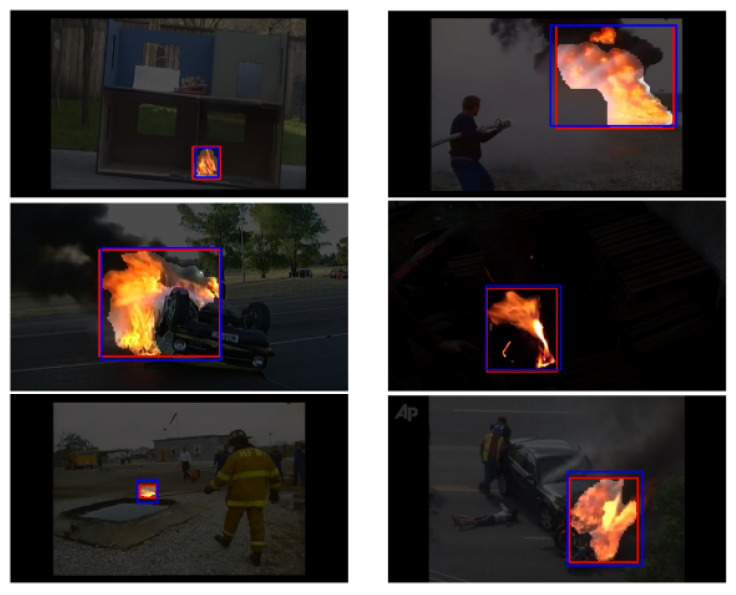
This figure illustrates qualitative results from the testing set. Red rectangle: prediction, blue rectangle: ground truth.

According to Table 1 and Table 2, the proposed method outperforms all the prior works, either on speed or on accuracy on the furg fire dataset [15]. This model has been evaluated for both full-frame classification tasks by considering that the frame contains fire if pixels with value 1 existed in the binary segmentation mask and fire localization task. In Table 1, the full-frame fire classification results are presented. All the metric values given from the proposed method, except of TPR and fps, are top compared to the prior works. More concretely, the FPR metric drops about 66%, compared to the next lowest value of that achieved via the InceptionV4-OnFire. F, P, and A are as high as the top results of NasNet-A-OnFire at 0.98, 0.99, and 0.97, respectively. The proposed method achieves top performance, as regards the speed, with a value at 20.4 fps, which is 13% higher than the ShuffleNetV2-OnFire model. The TPR metric has not been surpassed since 2011. In Table 2, the in-frame fire localization results are presented. As it seems, the proposed method outperforms the prior works in precision and similarity metrics. In more detail, P and S are 0.95, and they achieve an increase of 2% and 19% from the prior top results, respectively. However, TPR and F values are lower than the prior methods and more concretely about 23% and 11%, respectively, from the top method. This low TPR value is connected with the training dataset. As mentioned above, the dataset is not human-annotated, and this adds an error to the ground truths. In order to avoid the misclassification of non-fire pixels to fire pixels, and consequentially, a high FPR value, some fire pixels close to the fire boundaries were labeled as non-fire. So, the model learns to miss some of the fire pixels that are close to these boundaries. For improving the TPR metric for the in-frame localization problem, dilation is applied to the predicted segmentation mask. However, this dilation causes a reduction of the precision and similarity metrics. Finally, the proposed method is much faster than the two prior works in the in-frame localization problem, as can be seen in Table 1. Additionaly, Figure 8, depicts the model’s accurate localization performance on some sample frames from testing set.

#### 4.4.2. Full-Frame Classification Model

In Table 3, the quantitative results of the proposed method for the full-frame classification task and the state-of-the-art methods on the [25] dataset are presented. According to this, all the metrics given from the proposed method are surpassing the prior works, except the FPR metric. More concretely, the TPR and F metrics are 1% higher than the top prior works, the A and P metrics are equal to the top prior works, and the FPR metric is 33.3% higher than the top prior work. According to this, the proposed full-frame classification method is the only method that gives the highest values in almost all metrics.

## 5. Conclusions

In this work, a video fire detection method based on transformers is presented. It is a simplified architecture of DETR [14], and it is capable of recognizing fire in frames fast and accurately with a very low false positive rate. The paper presents a full-frame classification model that achieves top performance in almost all metrics on the [25] dataset and an in-frame localization model that achieves top performance in almost all metrics for the full-frame classification task on the [15] dataset. Moreover, it achieves top performance for precision and similarity metrics and top speed, compared to the prior works for the in-frame fire localization task on the [15] dataset. The creation of a human-annotated segmentation dataset with fire videos would increase the FPR metric in this work, but it would also be a big step for the research community in the field of fire detection in videos.

## Figures and Tables

**Table 1:** This table presents the quantitative results of the proposed method on the furg fire dataset [15], in comparison with prior works for the full-frame classification problem. The bolded values highlight each metric’s top value.

Models	TPR	FPR	F	P	A	fps
Chenebert, A., et al. [20]	**0.99**	0.28	0.92	0.86	0.89	0.16
InceptionV1-OnFire	0.92	0.17	0.90	0.88	0.89	8.4
InceptionV3-OnFire	0.94	0.07	0.94	0.93	0.94	13.8
InceptionV4-OnFire	0.94	0.06	0.94	0.94	0.94	12
NasNet-A-OnFire	0.98	0.15	0.98	0.99	0.97	5
ShuffleNetV2-OnFire	0.94	0.08	0.97	**0.99**	**0.97**	18
Ours	0.97	**0.02**	**0.98**	**0.99**	**0.97**	**20.4**

**Table 2:** This table presents the quantitative results of the proposed method on the furg fire dataset [15], in comparison with prior works for the in-frame fire localization problem. The bolded values highlight each metric’s top value.

Models	TPR	F	P	S
Chenebert, A., et al. [20]	**0.98**	**0.90**	0.93	0.80
InceptionV1-OnFire	0.92	0.88	0.84	0.78
Ours	0.75	0.80	**0.95**	**0.95**
Ours+dilation(3 × 3, 4iter)	0.78	0.81	**0.93**	**0.94**
Ours+dilation(3 × 3, 5iter)	0.79	0.81	**0.93**	**0.94**
Ours+dilation(3 × 3, 6iter)	0.80	0.82	**0.93**	**0.94**
Ours+dilation(3 × 3, 7iter)	0.80	0.82	0.92	**0.94**

**Table 3:** This table presents the quantitative results of the proposed method on the [25] dataset, in comparison with prior works for the full-frame fire classification problem. The bolded values highlight each metric’s top value.

Models	TPR	FPR	F	P	A
InceptionV1-OnFire	0.96	0.10	0.94	0.93	0.93
InceptionV3-OnFire	0.95	0.07	0.95	0.95	0.94
InceptionV4-OnFire	0.95	0.04	0.96	0.97	0.96
NasNet-A-OnFire	0.92	**0.03**	0.94	0.96	0.95
ShuffleNetV2-OnFire	0.93	0.05	0.94	0.94	0.95
Ours	**0.97**	0.04	**0.97**	**0.97**	**0.96**

## Data Availability

The datasets that were used for training and evaluation [15,25] can be found at https://collections.durham.ac.uk/files/r2d217qp536 (accessed on 3 November 2022).
